# Characterization of the Metabolic Fate of *Datura metel* Seed Extract and Its Main Constituents in Rats

**DOI:** 10.3389/fphar.2019.00571

**Published:** 2019-05-28

**Authors:** Cong Xia, Yan Liu, Hai Qi, Lulu Niu, Yuxuan Zhu, Wanying Lu, Xinyi Xu, Yongjian Su, Bingyou Yang, Qi Wang

**Affiliations:** ^1^Department of Medicinal Chemistry and Natural Medicine Chemistry, College of Pharmacy, Harbin Medical University, Harbin, China; ^2^Key Laboratory of Chinese Materia Medica, Heilongjiang University of Chinese Medicine, Harbin, China; ^3^Department of Cardiology, The 2nd Affiliated Hospital of Harbin Medical University, Harbin, China

**Keywords:** *Datura metel* seeds, metabolites identification, LC–MS fingerprinting, withanolides, amides, indoles

## Abstract

*Datura metel* L. has been frequently used in Chinese traditional medicine. However, little is known on the chemical composition and *in vivo* metabolism of its seeds. In this study, using the strategy “chemical analysis, metabolism of single representative compounds, and metabolism of extract at clinical dosage” that we propose here, 42 constituents were characterized from *D. metel* seeds water extract. Furthermore, the metabolic pathways of 13 representative bioactive compounds of *D. metel* seeds were studied in rats after the oral administration of *D. metel* seeds water extract at a clinical dosage (0.15 g/kg). These included three withanolides, two withanolide glucosides, four amides, one indole, one triterpenoid, one steroid, and one sesquiterpenoid, and with regard to phase II metabolism, hydroxylation, (de)methylation, and dehydrogenation reactions were dominant. Furthermore, the metabolism of *D. metel* seeds water extract provided to rats at a clinical dosage was investigated by liquid chromatography-tandem mass spectrometry based on the above metabolic pathways. Sixty-one compounds were detected in plasma, 83 in urine, and 76 in fecal samples. Among them, withanolides exhibited higher plasma exposure than the other types. To our knowledge, this is the first systematic study on the chemical profiling and metabolite identification of *D. metel* seeds, including all compounds instead of single constituents.

## Introduction

*Datura metel* L. (Solanaceae) seeds are one of the most popular herbal medicines for the treatment of rheumatoid arthritis and convulsions (Murthy et al., 2004). Although the seeds are toxic, they also have strong analgesic, anthelmintic, antioxidant, antimicrobial, antiviral, and antidiabetic activities (Wannang and Ndukwe, 2009; Kamaraj et al., 2011; Gu et al., 2014; Bachheti et al., 2018; Roy et al., 2018). Withanolides, flavonoids, alkaloids, sesquiterpenoids, lignans, and phenolic acids are generally considered the major bioactive compounds of *D. metel* (Kuang et al., 2008; Mai et al., 2017). However, there are few reports on the chemical composition of *D. metel* seeds. In previous studies, amides, indoles, sesquiterpenes, withanolides, and withanolide glucosides have been isolated from *D. metel* (Yang et al., 2010a, b; Bellila et al., 2011), but the constituents responsible for the treatment of different conditions have not been clarified.

It is well known that the *in vivo* metabolites of herbal medicines may play a substantial role in the therapeutics. Therefore, metabolites’ identification is critical for elucidating the bioactivities of complex herbal medicines. Recently, we studied the metabolism of two typological components, including two amides (*n*-trans-feruloyltyramine and cannabisin F) and two withanolide glucosides (daturataturin A and daturametelin I) of *D. metel* seeds in rats (Xu et al., 2018). To fully predict and identify their metabolites, we selected seven main types of representative compounds from the seeds.

**FIGURE 1:**
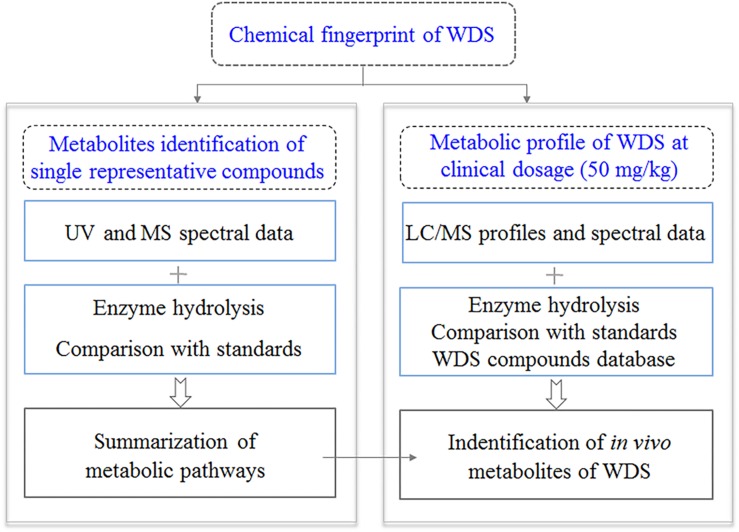
The three-step strategy proposed in the present study.

In this study, we aimed to improve this strategy so that it can be applied to other herbal medicines. Focusing on the identification of the *in vivo* metabolites of *D. metel* seed extracts after oral administration in rats, we first established the chemical fingerprint of *D. metel* seeds and identified 42 seed components (Figure 1). Second, we examined the *in vivo* metabolic pathways of seven groups of metabolites with different scaffolds, which included 13 representative compounds, using quadrupole time-of-flight mass spectrometry (qTOF-MS) and liquid chromatography-tandem mass spectrometry (LC/MS/MS). Finally, a normal clinical dosage of the herbal extract was administered to rats, and several metabolites were detected based on the metabolic pathways. Following this strategy, 113 metabolites (including 42 original phytochemicals and 71 newly formed ones) were detected at a clinical dosage (0.15 g/kg) in rats.

## Materials and Methods

### Chemicals and Reagents

**FIGURE 2:**
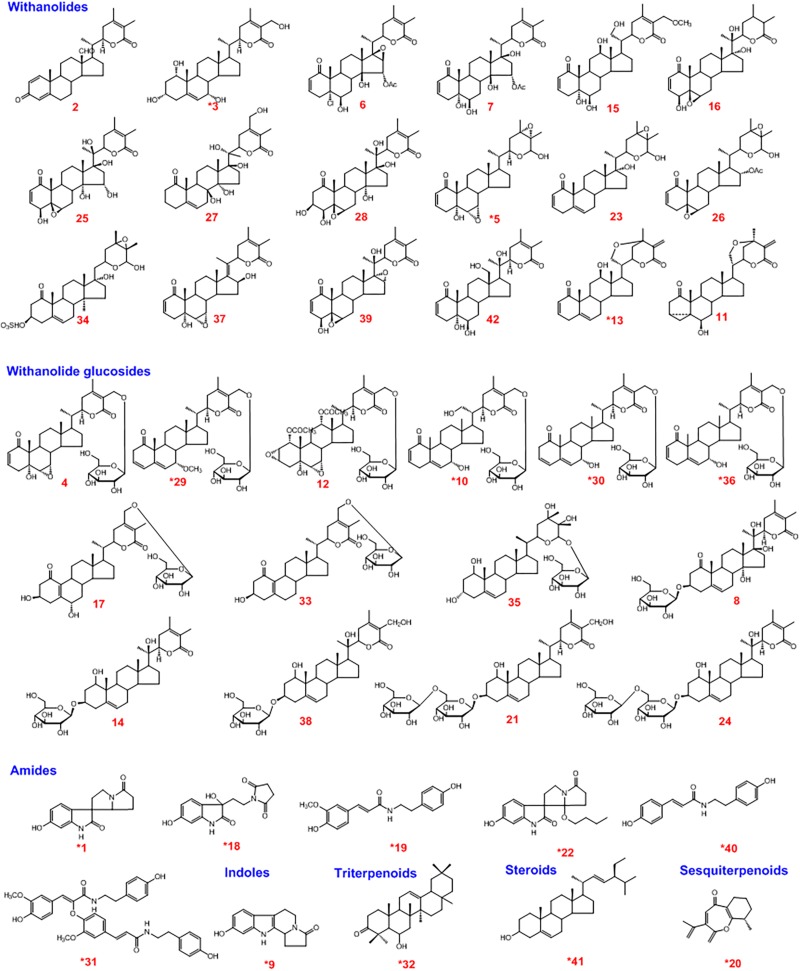
Structure of the chemical constituents of the water extract of *Datura metel* seeds (WDS).^*^Identified by comparing with reference standards.

The pure compounds daturametelindole B (**1**), daturametelin M (**3**), hyoscyamilactol (**5**), daturametelindole A (**9**), daturametelin L (**29**), daturametelindole D (**18**), *n*-trans-feruloyltyramine (**19**), daturametelindole C (**22**), daturametelin I (**30**), cannabisin F (**31**), daturaolone (**32**), daturataturin A (**36**), *n*-trans-p-coumaroyltyramine (**40**), and stigmasterol (**41**) were isolated from the seeds of *D. metel* by the authors. Their structures were characterized using nuclear magnetic resonance and MS (Figure 2 and Supplementary Figures S3–S22; Xu et al., 2018). Dinoxin B (**10**), withametelin L (**13**), and cinerolide (**20**) were purchased from Nantong Feiyu Biological Technology Co., Ltd. (Nanjing, China). Purities were above 98% according to high-performance liquid chromatography-ultraviolet (HPLC/UV) analysis. Beta-glucuronidase (HP-2 type, containing 100,000 U β-glucuronidase and 7500 U sulfatase per milliliter), β-nicotinamide adenine dinucleotide phosphate hydrate (β-NADP), d-glucose 6-phosphate sodium salt (G-6-P), and glucose-6-phosphate dehydrogenase (G-6-P-DE) used in the present study were purchased from Sigma–Aldrich (St. Louis, MO, United States). Heparin was purchased from HuiShi Biochemical Reagent Co., Ltd. (Shanghai, China). De-ionized water was prepared by a Milli-Q system (Millipore, Burlington, MA, United States). Acetonitrile, methanol, and formic acid (Sigma–Aldrich) were of HPLC grade. Other reagents were of analytical grade.

### Preparation of the Water Decoction of *Datura metel* Seeds (WDS)

*Datura metel* seeds were collected from the medicinal botanical garden of Heilongjiang University of Traditional Chinese Medicine. The authenticity of the sample was identified by Dr. Ruifeng Fan, Botanist, Department of Medicinal Plant, Heilongjiang University of Traditional Chinese Medicine. The voucher specimen (specimen number: 2016035) were preserved in the laboratory of Chinese Medicine Chemistry, Heilongjiang University of Traditional Chinese Medicine. The WDS was prepared by extracting 100 g of *D. metel* seeds decocted in 600 mL water three times (2.0, 2.0, 1.0 h). The decoctions were combined, filtered, and concentrated in vacuum at 50°C. The final concentration of the extract was 0.01 g/mL (crude drug per g/mL).

### Animals and Drug Administration

Male Sprague–Dawley rats (180–220 g) were purchased from the Laboratory Animal Center of the Second Affiliated Hospital of Harbin Medical University (Heilongjiang Province, China). The rats were kept in metabolic cages (465 mm × 300 mm × 200 mm), and the breeding room was at 25°C and 60 ± 5% relative humidity. All animals had free access to water and normal chow was provided *ad libitum* at a 12 h dark–light cycle for 3 days, and then fasted for 12 h before the experiments. The animal facilities and protocols were approved by the Animal Care and Use Committee of the Harbin Medical University. All procedures were in accordance with the National Institutes of Health Guide for the Care and Use of Laboratory Animals (ILAR, 1996).

The pure compounds, including **1**, **3**, **5**, **9**, **10**, **13**, **18**, **20**, **22**, **29**, **32**, **40**, and **41**, were suspended in 1% carboxy-methyl cellulose-sodium and were separately provided to rats (*n* = 4) orally at 20 mg/kg. The WDS was provided to rats (*n* = 4) at 0.5 g/kg (clinical dosage, equivalent to 600 mg/day for a 60-kg human), respectively. The dosage in our study was the clinically safe and effective (Pharmacopoeia Commission, 2015). The control group (*n* = 4) was administrated with 1000 μL normal saline.

### Preparation of Plasma, Urine, and Fecal Samples

To obtain plasma samples, blood (1000 μL) was collected from the angular vein into heparinized tubes at six time points: 0.5, 1, 2, 4, 6, and 10 h after the administration of WDS and pure compounds (*n* = 4). The blood of two rats was collected at 0.5, 2, and 6 h, or at 1, 4, and 10 h, and then centrifuged at 10,000 × *g* for 10 min at 4°C to obtain the plasma and then pooled plasma samples of different time points. Urine and fecal samples were collected for 0–24 h from rats held in metabolic cages (DXL-D, Keke Medical Model Co., Ltd., Shanghai, China).

Plasma, urine, and fecal samples were prepared as described in our previous report (Xu et al., 2018). The residue of these samples was dissolved in 300 μL of methanol, and filtered through a 0.22-μm membrane for ultra-high performance liquid chromatography coupled with electrospray ionization (UPLC/ESI) qTOF-MS analysis.

### Incubation of Rat Liver Microsomes

Compounds **3**, **5**, **10**, **13**, **29**, **40**, and **41** were separately dissolved in methanol, and then diluted with phosphate buffered saline (PBS). The final concentration of each compound in the 200-μL incubation mixture (NADPH-generating system, 100 mM potassium phosphate buffer (pH 7.4), and rat liver microsomes) was 25 μM, and the amount of organic solvent in the mixture was lower than 1% (v/v). PBS-containing methanol was added as the negative control. The incubation was conducted at 37°C for 2 h. After this period, the reaction was terminated by adding 1000 μL of cold acetonitrile. The mixture was then kept at 4°C for 30 min, and the precipitated protein was removed by centrifugation at 10,000 × *g* for 10 min at 4°C.

### Enzyme Hydrolysis

A 100-μL aliquot of plasma or urine sample was dried under a gentle flow of nitrogen gas and then mixed with 400 μL of b-glucuronidase solution (containing 19.86 U/μL, in sodium acetate buffer, pH 5.5). The mixture was vortexed for 5 min, incubated in a 37°C water bath for 1.5 h, treated with 1000 μL of cold methanol–acetonitrile (1:1, v/v) to precipitate the protein, and then centrifuged at 10,000 × *g* for 10 min at 4°C. The supernatant was dried under a gentle nitrogen flow and then dissolved in 100 μL of methanol. The solution was filtered through a 0.22-μm membrane for analysis.

### UPLC/ESI/qTOF-MS Analysis

The analysis was performed on an ACQUITY UPLC instrument coupled with a Xevo G2 qTOF mass spectrometer (Waters, Milford, MA, United States) via an ESI ion source. The autosampler was controlled via the MassLynx^TM^ 4.1 software (Waters). Samples were separated on an ACQUITY UPLC HSS T3 column (2.1 mm × 100 mm, 1.7 μm; Waters). The mobile phase consisted of acetonitrile (A) and water containing 0.1% (v/v) formic acid (B) at a flow rate of 400 μL/min. The gradient elution program was set as follows: 0–7 min, 10–22% A; 7–13 min, 22–70% A; 13–17 min, 70–90% A; 17–20 min, 90–95% A; 20–22 min, 95% A. A 2-μL sample aliquot was injected for analysis, and the column temperature was 40°C.

The qTOF/MS system was equipped with an ESI source operating in positive ion mode, as in our previous report (Xu et al., 2018). High-purity nitrogen (N_2_) and high-purity helium (He) were used as desolvation gas and collision gas, respectively. The flow rate of N_2_ was 600 L/h and that of He was 50 L/h. The optimized parameters were: capillary voltage, 3.5 kV; sample cone voltage, 30 V; and extraction cone voltage, 4 V. The desolvation and source temperatures were 350°C and 100°C, respectively. The MS full scan range was 100–1000 *m*/*z*, and MS^n^ range was 100–800 *m*/*z*. All data collected in the positive ion mode were acquired and processed by the MassLynx^TM^ 4.1 software (Waters). UHPLC/qTOF-MS is the high-resolution mass spectra, which could provide the accurate [M+H]^+^ ions of the metabolites.

### Quadrupole Ion Trap (Q TRAP) LC/MS/MS

The AB SCIEX 4000 Q TRAP^TM^ composite triple quadrupole/linear ion trap tandem mass spectrometer (SCIEX, Framingham, MA, United States) connected to the UHPLC via the ESI interface (Applied Biosystems, Foster City, CA, United States) was operated in the positive ion mode. The column effluent was split using a zerodead-volume “T” connector, with approximately half of the flow being fed to the mass spectrometer. The interface and parameters of the mass spectrometer were as follows: spray capillary voltage, 5.5 kV; DP, 70 V; EP, 0 V; CE, 45 V; nebulizer pressure, 40 psi; dry gas pressure, 40 psi; curtain gas pressure, 10 psi; and dry gas temperature, 600°C. All data were acquired and processed using Analyst (SCIEX). The mass spectrum fragment ions of MS^2^ and MS^3^ could be obtained by Q-TRAP LC/MS/MS.

## Results and Discussion

### Characterization of the Chemical Constituents of WDS

**FIGURE 3:**
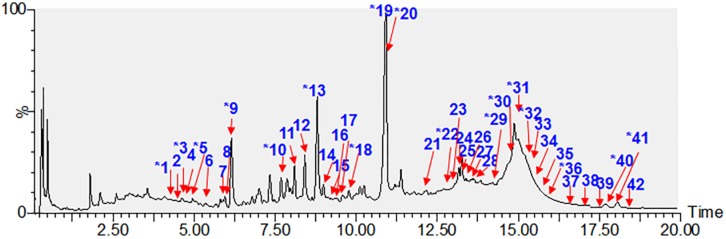
LC/MS total ion currents of the water extract of *Datura metel* seeds (WDS). ^*^Identified by comparing with reference standards.

A rapid and sensitive UHPLC/qTOF-MS method was employed to characterize the chemical constituents of WDS. Forty-two compounds (**1–42**) were identified or tentatively characterized (Figure 3), including 18 withanolides, 14 withanolide glucosides, six amides, one indole, one triterpenoid, one steroid, and one sesquiterpenoid. Among them, 17 compounds (**1**, **3**, **5**, **9**, **10**, **13**, **18**, **19**, **20**, **22**, **29**, **30**, **31**, **32**, **36**, **40**, and **41**) were unambiguously identified by a comparison with reference standards. Another 25 potential constituents (**2**, **4**, **6**, **7**, **8**, **11**, **12**, **14**, **15**, **16**, **17**, **21**, **23**, **24**, **25**, **26**, **27**, **28**, **33**, **34**, **35**, **37**, **38**, **39**, and **42**) were tentatively characterized based on their retention times, UV spectra, high-accuracy mass spectra, and MS/MS fragmentation behaviors. Chemical structures of the single compounds characterized in WDS are given in Figure 2.

### Metabolic Pathways of Single Representative Compounds

The chemical constituents of WDS were classified into seven groups according to their structural type: withanolides (A), withanolide glucosides (B), amides (C), indoles (D), triterpenoids (E), steroids (F), and sesquiterpenoids (G). In our previous study, we selected four representative compounds containing two withanolide glucosides (daturataturin A and daturametelin I), and two amides (cannabisin F and *n*-trans-feruloyltyramine) from WDS and elucidated their metabolic pathways (Xu et al., 2018). In the present study, we used these pathways for metabolite identification of other compounds, which had similar scaffolds in WDS, developing a “compound to extract” strategy. The metabolic pathways of daturametelindole B (**1**), daturametelin M (**3**), hyoscyamilactol (**5**), daturametelindole A (**9**), dinoxin B (**10**), withametelin L (**13**), daturametelindole D (**18**), cinerolide (**20**), daturametelindole C (**22**), daturametelin L (**29**), daturaolone (**32**), *n*-trans-p-coumaroyltyramine (**40**), and stigmasterol (**41**) were first reported in this study, and these 13 single compounds were selected to represent seven major scaffolds in WDS. These compounds were labeled in the HPLC fingerprint of WDS. As shown in Table 1, the multistage mass spectrum fragmentation information (ESI-MS^n^) was obtained both from UPLC/ESI/qTOF-MS and Q TRAP LC/MS/MS analysis.

**TABLE 1:** Characterization of *in vivo* metabolites of *Datura metel* seeds by UPLC/ESI/qTOF-MS and Q-Trap LC/MS/MS in this study.

**No.**	**RT**	**Formula**	**HR-MS [M+H]^+^**			**(+)ESI-MS^n^(*m*/*z*)**	**Metabolic Reaction**	**Plasma**	**Urine**	**Feces**	
	**(min)**		**Measured**	**Predicted**	**Δ (ppm)**						**Source**
^*^1	4.32	C_14_H_14_N_2_O_3_	259.1072	259.1077	1.9	MS^2^[259]: 240,162,150,114	Daturametelindole B	–	–	+	D^S^,D^W^
^a^ 1-M1	3.17	C_20_H_22_N_2_O_9_	453.1405	453.1398	–1.5	MS^2^[453]: 340,150	+GluA	+	++	++	D^S^,D^W^
						MS^3^[150]: 114					
1-M2	3.68	C_14_H_14_N_2_O_6_S	339.0652	339.0645	–2.1	MS^2^[339]: 321,150	+Sul	++	+	–	D^S^,D^W^
						MS^3^[150]: 122,114					
1-M3	5.28	C_14_H_12_N_2_O_3_	257.0913	257.0921	3.1	MS^2^[257]: 150,136,122	−2H	–	+	–	D^S^
**2**	4.48	C_28_H_36_O_4_	437.2689	437.2686	–0.7	MS^2^[437]: 283,258,240,150	Paraminabeolide A	+	+	–	D^W^
^*^3	4.52	C_28_H_42_O_6_	475.3022	475.3054	6.7	MS^2^[475]: 305,135,170	Daturametelins M	+	+	–	D^S^,D^W^
3-M1	4.03	C_28_H_40_O_5_	457.2956	457.2949	–1.5	MS^2^[457]: 287,163,149	-H_2_O	+	+	++	D^S^,D^W^
3-M2	4.24	C_28_H_44_O_8_	509.3112	509.3109	–0.6	MS^2^[509]: 340,136	+H_2_O+OH	+	+	++	D^S^,D^W^
3-M3	4.36	C_28_H_42_O_7_	491.3001	491.3003	–0.4	MS^2^[491]: 323,155,135	+OH	+	+	–	D^S^,D^W^
3-M4	4.42	C_29_H_44_O_6_	489.3229	489.3212	–3.4	MS^2^[489]: 319,136	+CH_3_	–	+	+	D^S^
**4**	4.54	C_34_H_48_O_11_	633.3274	633.3269	–0.8	MS^2^[633]: 469,453,203,150	Baimantuoluoside C	+	–	–	D^W^
4-M1	3.69	C_33_H_46_O_14_S	699.2673	699.2681	1.1	MS^2^[699]: 619,455	+Sul-CH_3_	+	+	–	D^W^
						MS^3^[455]: 273,187,163,136					
4-M2	4.18	C_34_H_46_O_11_	631.3119	631.3113	–1.0	MS^2^[631]: 467,288,136	−2H	–	++	–	D^W^
4-M3	4.35	C_40_H_56_O_17_	809.3598	809.3590	–0.9	MS^2^[809]: 633,469,295,136	+GluA	–	++	–	D^W^
^*^5	4.60	C_28_H_40_O_6_	473.2887	473.2898	2.3	MS^2^[473]: 301,206,171	Hyoscyamilactol	+	+	++	D^S^,D^W^
5-M1	4.13	C_28_H_40_O_7_	489.2838	489.2847	1.8	MS^2^[489]: 338,317,171,135	+OH	++	+	+	D^S^,D^W^
**6**	5.38	C_30_H_39_ClO_8_	563.2412	563.2406	–1.0	MS^2^[563]: 409,393,260,153	Physagulin I	+	+	+	D^W^
6-M1	4.31	C_30_H_39_ClO_11_S	643.1985	643.1974	–1.7	MS^2^[643]: 563,409,164	+Sul	–	+	+	D^W^
6-M2	4.95	C_36_H_45_ClO_14_	737.2583	737.2571	–1.6	MS^2^[737]: 561,407,150	+GluA-2H	–	–	+	D^W^
**7**	5.86	C_30_H_42_O_9_	547.2879	547.2902	4.2	MS^2^[547]: 393,240,153,114	Physagulin K	–	+	+	D^W^
**8**	5.92	C_34_H_50_O_12_	651.3371	651.3375	0.6	MS^2^[651]: 487,319,288,163	Coagulin L	+	+	–	D^W^
^*^9	6.12	C_14_H_14_N_2_O_2_	243.1116	243.1128	4.9	MS^2^[243]: 215,187,107	Daturametelindole A	+	+	+	D^S^,D^W^
^a^ 9-M1	2.75	C_20_H_20_N_2_O_8_	417.1297	417.1292	–1.2	MS^2^[417]: 241,150,134	+GluA-2H	–	–	++	D^S^,D^W^
^a^ 9-M2	4.69	C_20_H_22_N_2_O_9_	435.1388	435.1398	2.3	MS^2^[435]: 259,231,136	+GluA+OH	++	+	–	D^S^,D^W^
9-M3	5.28	C_15_H_16_N_2_O_2_	257.1296	257.1285	–4.2	MS^2^[257]: 243,215,136,107	+CH_3_	+	+	++	D^S^,D^W^
^a^ 9-M4	5.82	C_20_H_22_N_2_O_11_S	499.1002	499.1017	3.0	MS^2^[499]: 419,243,150	+GluA+Sul	+	+	–	D^S^,D^W^
9-M5	6.49	C_13_H_12_N_2_O_5_S	309.0532	309.0540	2.5	MS^2^[309]: 229,136,122	+Sul-CH_3_	++	+	–	D^S^,D^W^
9-M6	6.68	C_14_H_14_N_2_O_6_S	339.0652	339.0645	–2.0	MS^2^[339]: 259,243,215	+Sul+OH	++	++	–	D^S^,D^W^
^*^10	7.52	C_34_H_48_O_11_	633.3244	633.3269	3.9	MS^2^[633]: 469,437	Dinoxin B	+	++	+	D^s^, D^w^
						MS^3^[469]: 285,136				
10-M1	5.88	C_34_H_48_O_12_	649.3251	649.3219	–4.9	MS^2^[649]: 483,299,136	+OH	+	++	+	D^S^,D^W^
10-M2	6.03	C_35_H_50_O_11_	647.3442	647.3426	–2.4	MS^2^[647]: 483,471,285	+CH_3_	+	+	++	D^S^,D^W^
^a^ 10-M3	7.06	C_40_H_56_O_17_	809.3586	809.3590	0.5	MS^2^[809]: 437,469,246,136	+GluA	+	++	++	D^S^,D^W^
10-M4	8.44	C_34_H_46_O_11_	631.3132	631.3113	–3.0	MS^2^[632]: 467,421,228,114	-2H	+	+	+	D^S^,D^W^
10-M5	9.78	C_28_H_35_O_6_	468.2533	468.2506	–5.7	MS^2^[468]: 283,134	-Glc-2H	–	+	+	D^S^,D^W^
**11**	8.14	C_28_H_38_O_5_	455.2799	455.2792	–1.5	MS^2^[455]: 287,167,152,135	Withawrightolide	–	–	+	D^W^
**12**	8.36	C_38_H_54_O_15_	751.3517	751.3535	2.3	MS^2^[751]: 589,419,331,193	Chantriolide C	–	–	+	D^W^
12-M1	8.51	C_44_H_62_O_21_	927.3855	927.3856	0.1	MS^2^[927]: 751,589,419	+GluA	–	–	++	D^w^
						MS^3^[419]: 331,193,155,134					
12-M2	7.58	C_37_H_52_O_18_S	817.2951	817.2947	–0.5	MS^2^[817]: 737,575,405,193	+Sul-CH_3_	–	–	+	D^W^
12-M3	7.62	C_38_H_56_O_17_	785.3573	785.3590	2.1	MS^2^[785]: 391,357,193,163	+OH+H_2_O	–	–	++	D^W^
12-M4	8.00	C_39_H_56_O_16_	781.3628	781.3641	1.6	MS^2^[781]: 391,373,355,193,163	+OH+CH_3_	–	–	++	D^W^
12-M5	14.98	C_38_H_52_O_15_	749.3361	749.3379	2.4	MS^2^[749]: 357,283,193,163	-2H	–	–	++	D^W^
^*^13	8.73	C_28_H_36_O_5_	453.2654	453.2636	–3.9	MS^2^[453]: 341,285,167,114	Withametelin L	++	++	++	D^S^,D^W^
13-M1	7.74	C_27_H_34_O_8_S	519.2055	519.2047	–1.5	MS^2^[519]: 439,271,167,150	+Sul-CH_3_	+	+	+	D^S^,D^W^
13-M2	8.19	C_28_H_36_O_6_	469.2574	469.2585	2.3	MS^2^[469]: 301,269,167	+OH	++	++	++	D^S^,D^W^
**14**	8.91	C_34_H_52_O_10_	621.3642	621.3633	–1.4	MS^2^[621]: 509,457	Coagulin Q	+	+	+	D^w^
						MS^3^[457]: 314,287,163,135					
**15**	9.05	C_29_H_42_O_8_	519.2957	519.2950	–1.3	MS^2^[519]: 319,295,262,199,165	Baimantuoluoline C	+	+	+	D^W^
15-M1	7.56	C_29_H_42_O_9_	535.2914	535.2902	–2.2	MS^2^[535]: 509,335,199,165	+OH	++	+	+	D^W^
**16**	9.40	C_28_H_40_O_7_	489.2856	489.2847	–1.8	MS^2^[489]:439,317,353,171	Philadelphicalactone A	–	+	+	D^W^
**17**	9.43	C_33_H_48_O_11_	621.3288	621.3269	–3.0	MS^2^[621]: 457,289,136	Withalongolide L	+	++	–	D^W^
17-M1	7.37	C_33_H_48_O_14_S	701.2834	701.2838	–0.5	MS^2^[701]: 621,457,289,136	+Sul	–	++	++	D^W^
^*^18	9.77	C_14_H_14_N_2_O_5_	291.0968	291.0975	2.4	MS^2^[291]: 246,219,155,135	Daturametelindole D	+	+	++	D^S^,D^W^
18-M1	7.48	C_13_H_12_N_2_O_8_S	357.0391	357.0387	–1.1	MS^2^[357]: 277,144,135	+Sul-CH_3_	+	+	++	D^S^
18-M2	8.43	C_14_H_14_N_2_O_8_S	371.0538	371.0544	1.6	MS^2^[371]: 291,155,135	+Sul	–	+	++	D^S^,D^W^
^*^19	10.86	C_18_H_19_NO_4_	314.1366	314.1387	6.6	MS^2^[314]: 314,177,163	N-trans-feruloyltyramine	–	–	++	D^W^
^a^ 19-M1	8.50	C_24_H_27_NO_11_	506.1669	506.1657	–2.3	MS^2^[506]: 330,314,177,145	+GluA+OH	++	+	–	D^W^
19-M2	8.14	C_18_H_19_NO_5_	330.1339	330.1336	–0.9	MS^2^[330]: 177,137	+OH	++	+	–	D^W^
19-M3	9.65	C_18_H_17_NO_4_	312.1232	312.1230	–0.6	MS^2^[312]: 177,145,114	-2H	++	++	++	D^W^
^*^20	10.91	C_15_H_18_O_2_	231.1377	231.1380	1.3	MS^2^[231]: 215,203,190,136	Cinerolide	+	+	++	D^S^,D^W^
^a^ 20-M1	6.46	C_21_H_24_O_8_	405.1552	405.1544	–1.9	MS^2^[405]: 229,187,201,134	+GluA-2H	+	+	–	D^S^,D^W^
20-M2	8.60	C_15_H_18_O_5_S	311.0934	311.0948	4.5	MS^2^[311]: 231,190,136	+Sul	++	+	–	D^S^,D^W^
20-M3	9.92	C_14_H_16_O_2_	217.1211	217.1223	5.5	MS^2^[217]: 189,174	-CH_3_	+	+	–	D^S^
**21**	12.10	C_40_H_62_O_15_	783.4153	783.4161	1.0	MS^2^[783]: 457,355,325,288	Withanoside IV	–	+	–	D^W^
21-M1	8.06	C_41_H_64_O_16_	813.4274	813.4267	–0.8	MS^2^[814]: 487,325,318,226,288	+OH+CH_3_	–	++	++	D^W^
^*^22	12.87	C_18_H_22_N_2_O_4_	331.1660	331.1652	–2.4	MS^2^[331]: 325,223,189,107	Daturametelindole C	++	+	–	D^S^,D^W^
22-M1	8.21	C_17_H_20_N_2_O_7_S	397.1068	397.1064	–1.0	MS^2^[397]: 317,209,107	+Sul-CH_3_	++	++	++	D^S^,D^W^
22-M2	10.07	C_17_H_20_N_2_O_4_	317.1487	317.1496	2.8	MS^2^[317]: 209,150,107	-CH_3_	++	+	–	D^S^,D^W^
**23**	12.93	C_28_H_40_O_5_	457.2945	457.2949	0.8	MS^2^[457]: 285,171,151,147	Cilistol A	–	–	+	D^W^
23-M1	8.96	C_34_H_48_O_11_	633.3257	633.3269	1.9	MS^2^[633]: 457,171,151	+GluA	–	–	+	D^W^
**24**	13.09	C_40_H_62_O_15_	783.4154	783.4161	0.9	MS^2^[783]: 457,325,289,162	Withanoside VI	–	–	+	D^W^
**25**	13.21	C_28_H_38_O_9_	519.2577	519.2589	2.3	MS^2^[520]: 349,197,137	Withangulatin B	–	–	+	D^W^
**26**	13.41	C_30_H_42_O_7_	515.3018	515.3003	–2.9	MS^2^[515]: 343,329,193,171,150	Virginol C	–	+	+	D^W^
26-M1	15.29	C_36_H_50_O_14_	707.3282	707.3273	–1.2	MS^2^[708]: 532,359,193,171,166	+GluA+OH	–	++	++	D^W^
**27**	13.48	C_28_H_40_O_8_	505.2786	505.2796	1.9	MS^2^[505]: 319,264,185,136	Ajugin D	+	–	–	D^W^
27-M1	10.24	C_34_H_48_O_17_S	761.2674	761.2685	1.4	MS^2^[761]: 585,505,319,185	+GluA+Sul	–	–	++	D^W^
**28**	13.98	C_28_H_40_O_9_	521.2747	521.2745	–0.4	MS^2^[521]: 351,255,184,169	Phyperunolide E	–	–	+	D^W^
28-M1	4.63	C_34_H_48_O_15_	697.3059	697.3066	1.0	MS^2^[697]: 521, 351,203,169	+GluA	–	+	–	D^W^
28-M2	14.5	C_27_H_38_O_9_	507.2578	507.2589	2.1	MS^2^[508]: 351,184,167,155	-CH_3_	–	+	–	D^W^
^*^29	14.11	C_35_H_50_O_10_	631.3441	631.3477	5.7	MS^2^[631]: 566,467	Daturametelin L	–	+	+	D^s^, D^w^
						MS^3^[467]: 299,162,136					
29-M1	9.60	C_35_H_50_O_11_	647.3439	647.3426	–2.0	MS^2^[647]: 483,469,285,135	+OH	+	+	–	D^S^,D^W^
^a^ 29-M2	10.61	C_41_H_58_O_16_	807.3781	807.3798	2.1	MS^2^[807]: 631,453,285,135	+GluA	–	++	++	D^S^,D^W^
^*^29-M3	14.93	C_34_H_48_O_10_	617.3315	617.3320	0.8	MS^2^[617]: 453,441,285,135	-CH_3_ (**30**)	–	++	+	D^W^
29-M4	15.15	C_29_H_37_O_5_	466.2720	466.2714	–1.2	MS^2^[466]: 453,285,134	-Glc-2H	–	++	++	D^W^
^*^30	14.93	C_34_H_48_O_10_	617.3312	617.3320	1.3	MS^2^[617]: 453, 285,271,135	Daturametelin I	–	++	+	D^W^
^a^ 30-M1	4.53	C_34_H_48_O_11_	633.3242	633.3269	4.2	MS^2^[633]: 469,285,136	+OH	–	+	+	D^W^
^*^31	15.08	C_36_H_36_N_2_O_8_	625.2521	625.2544	3.6	MS^2^[625]:488,377,298,164,136	Cannabisin F	–	+	++	D^W^
^a^ 31-M1	8.83	C_42_H_44_N_2_O_15_	817.2854	817.2814	–4.8	MS^2^[817]:641,611,488,373,298	+GluA+OH	–	+	++	D^W^
31-M2	9.28	C_35_H_34_N_2_O_8_	611.2368	611.2388	3.2	611,474,373,298,136	-CH_3_	+	+	++	D^W^
^*^32	15.21	C_30_H_48_O_2_	441.3712	441.3727	3.3	MS^2^[441]: 423,357,150	Daturaolone	–	–	++	D^S^,D^W^
^a^ 32-M1	7.54	C_36_H_56_O_9_	633.3999	633.3997	–0.3	MS^2^[633]: 457,435,114	+GluA+OH	+	–	–	D^S^
**33**	15.35	C_33_H_48_O_10_	605.3317	605.3320	0.4	MS^2^[605]: 441,273,148,138	Withalongolide M	–	–	+	D^W^
33-M1	7.10	C_33_H_48_O_14_S	701.2854	701.2838	–2.2	MS^2^[701]: 621,457,289,135	+Sul+OH	++	+	–	D^W^
**34**	15.39	C_28_H_42_O_9_S	555.2606	555.2622	2.8	MS^2^[555]: 357,299,150	Cilistol Y	+	+	+	D^W^
**35**	15.53	C_34_H_56_O_11_	641.3874	641.3895	3.2	MS^2^[642]: 429,385,341,150	Cilistol J	–	–	+	D^W^
^*^36	15.62	C_34_H_48_O_10_	617.3315	617.3320	0.8	MS^2^[617]: 453,285,136	Daturataturin A	–	+	+	D^W^
^*^36-M1	7.53	C_34_H_48_O_11_	633.3247	633.3269	3.4	MS^2^[633]: 469,285,136	+OH (**10**)	++	+	+	D^W^
^a^ 36-M2	7.82	C_40_H_54_O_16_	791.3451	791.3485	4.2	MS^2^[791]: 617,451,268,133	+GluA-2H	+	–	–	D^W^
36-M3	9.52	C_35_H_50_O_10_	631.3496	631.3477	–3.0	MS^2^[631]: 453,299,136	+CH_3_	+	+	–	D^W^
36-M4	11.63	C_28_H_36_O_5_	453.2653	453.2636	–3.7	MS^2^[454]: 437,283,136	-Glc-2H	+	++	++	D^W^
**37**	16.68	C_28_H_36_O_6_	469.2565	469.2585	4.2	MS^2^[469]: 316,270,153,136,122	Exodeconolide A	+	+	+	D^W^
**38**	17.03	C_34_H_52_O_11_	637.3568	637.3582	2.1	MS^2^[637]: 457,285,185,153	Withanoside XI	–	++	++	D^W^
38-M1	8.03	C_40_H_60_O_17_	813.3912	813.3903	–1.1	MS^2^[813]: 637,457,285,185	+GluA	++	++	++	D^W^
**39**	17.58	C_28_H_36_O_7_	485.2521	485.2534	2.6	MS^2^[485]: 315,176,169,151	20-Hydroxytubocapsanolide A	–	–	+	D^W^
^*^40	17.74	C_17_H_17_NO_3_	284.1286	284.1281	–1.7	MS^2^[284]: 147,136,122	*n*-trans-p-coumaroyltyramine	++	++	++	D^S^,D^W^
40-M1	10.96	C_17_H_19_NO_5_	318.1358	318.1336	–6.9	MS^2^[318]: 274,230,150,114	+OH+H_2_O	++	++	++	D^S^,D^W^
40-M2	12.93	C_17_H_17_NO_4_	300.1211	300.1230	6.3	MS^2^[300]: 152,114	+OH	++	+	+	D^S^,D^W^
^a^ 40-M3	13.24	C_23_H_25_NO_9_	460.1623	460.1602	–4.5	MS^2^[460]:284,136	+GluA	+	++	++	D^S^,D^W^
40-M4	14.09	C_17_H_17_NO_6_S	364.0833	364.0849	4.3	MS^2^[364]:284,147,136	+Sul	+	++	++	D^S^,D^W^
40-M5	16.33	C_23_H_23_NO_9_	458.1437	458.1446	1.9	MS^2^[458]: 282,147,134	+GluA-2H	–	+	++	D^S^,D^W^
^*^41	18.08	C_29_H_48_O	413.3790	413.3778	–2.9	MS^2^[413]: 273,139,135	Stigmasterol	++	++	–	D^S^,D^W^
41-M1	8.74	C_29_H_48_O_2_	429.3741	429.3727	–3.2	MS^2^[429]: 289,274,139	+OH	++	+	–	D^S^,D^W^
41-M2	12.08	C_29_H_46_O_3_	443.3509	443.3520	2.4	MS^2^[443]: 289,196,153,141,135	+2OH	+	+	–	D^S^,D^W^
41-M3	13.42	C_29_H_50_O_3_S	479.3563	479.3553	–2.0	MS^2^[479]: 399,275,137	+2H+Sul	–	+	++	D^S^,D^W^
**42**	18.43	C_28_H_40_O_7_	489.2821	489.2847	5.3	MS^2^[489]: 319,169,151	Jaborosalactone VIII	+	–	–	D^W^

^*^,Identified by comparing with reference standards; ++, detected at high abundance, > 1 × e^4^; +, only detected, 1 × e^3^ ∼ 1 × e^4^; –, not detected. ^a^ Confirmed by enzyme hydrolysis. D^S^, detected in single compound administration biosamples; D^W^, detected in WDS administration biosamples.

#### Withanolides

Compounds **3**, **5**, and **13** were chosen as representatives of withanolides (Figure 2). They were considered as bioactive constituents (Ma et al., 1999; Zhang et al., 2018) and could be detected *per se* in plasma and urine samples. Hydroxylation was the major metabolic reaction for withanolides from *D. metel* (Table 1). In addition, compounds **3** and **5** may be metabolized into 21-OH daturametelin M and 21-OH hyoscyamilactol, respectively. This reaction was also observed for daturataturin A (**36**) and daturametelin I (**30**) in our previous study (Xu et al., 2018). However, the hydroxyl position of **13** could not be assigned due to the limited structural information. Hydroxylated products were also detected when **3**, **5**, and **13** were incubated in rat liver microsomes, indicating that the hydroxylation reaction was catalyzed by P450 enzymes (Supplementary Figure S1).

#### Withanolide Glucosides

**FIGURE 4:**
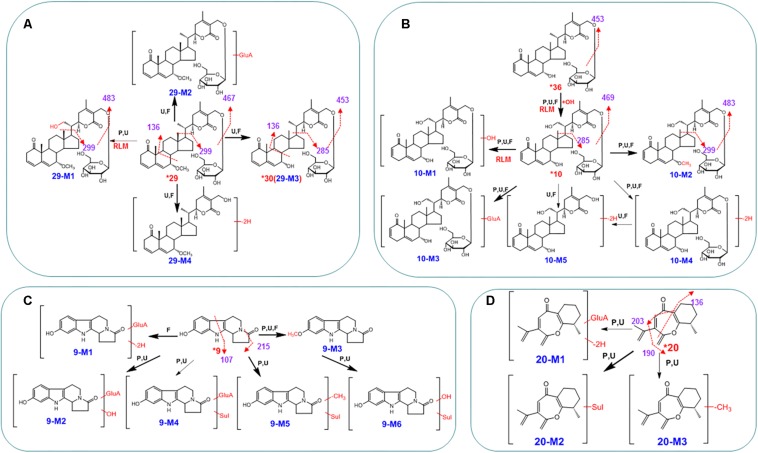
The proposed metabolic pathways for compounds **9** **(C)**, **10** **(B)**, **20** **(D)**, and **29** **(A)** in rats after the oral administration of the WDS. Red bold arrows indicate major metabolites; ^*^, compared with reference standards; U, detected in urine samples; P, detected in plasma samples; F, detected in fecal samples; RLM, detected in rat liver microsomes; Sul, sulfate; GluA, glucuronic acid residue.

The metabolism of two withanolide glucosides, **10** and **29**, was investigated. When *O*-glycosides lose the sugar residue to produce corresponding aglycones (withanolides), dehydrogenation occurs. This reaction of interconversion was very common for withanolide glucosides. In addition, hydroxylation, (de)methylation, and glucuronidation of withanolide glucosides were also major reactions as shown in Figures 4A,B. These metabolites, derived from the three withanolide glucosides, were detected in plasma, urine, and fecal samples (Table 1). For example, **29** produced the hydroxylated metabolite **29-M1** (*m*/*z* 647→483, 469, 299, 136), the demethylated metabolite **29-M3** (*m*/*z* 617→453, 285, 136, identified as **30**), the glucuronide-conjugated metabolite **29-M2** (*m/z* 807→631, 453, 285, 136), and hydrolyzed and dehydrogenated metabolite **29-M4** (*m/z* 466→453, 285, 134).

#### Amides

Amides are abundant in *D. metel* seeds, and considered as characteristic components (Karim et al., 2017). Four major amides (**1**, **18**, **22**, and **40**) were chosen to examine the *in vivo* metabolism of this type of compounds. In brief, the metabolism of amides varied significantly according to the group substitutions on their backbones. For example, **1** and **40** could undertake different phase I reactions, including hydroxylation, dehydrogenation, hydroxylation, and hydration. Phase II conjugation reactions (to form glucuronides and sulfates) were common in amides (Table 1), but the peaks of glucuronides disappeared when the sample was treated with β-glucuronidase (Supplementary Figure S2). Amides **18** and **22** were mainly involved in demethylation and sulfation. The major metabolic reactions and metabolite distributions of these four amides are listed in Table 1. Plasma and urine samples mainly contained phase II metabolites and hydroxylated products, while urine samples comprised most metabolites.

#### Indoles

Indole alkaloids in *D. metel* seeds are extremely important, and their distribution and metabolism have been reported in several different organisms (Gillam et al., 2000; van der Fits and Memelink, 2000; Rischer et al., 2006; Ziegler and Facchini, 2008). We chose daturametelindole A (**9**) for examining metabolic pathways. The qTOF mass spectra showed [M+H]^+^ ions at *m*/*z* 243.1116, consistent with the molecular formula of C_14_H_14_N_2_O_2_. In the ion trap MS^n^ spectra, the [M+H]^+^ ions could further add a glucuronic acid moiety (176 U), and then be dehydrogenized, hydroxylated, and sulfated to produce the corresponding ions at *m*/*z* 417 (**9-M1**), 435 (**9-M2**), and 499 (**9-M4**), respectively (Figure 4C). The above conjugates were confirmed by enzyme hydrolysis. When indole **9** from the plasma sample was treated with β-glucuronidase, the peaks of **9-M1**, **9-M2**, and **9-M4** disappeared, and the peak corresponding to **9** increased remarkably (Figure 4C). Thus, it could be deduced that these metabolites were glucuronides of **9**. The sulfate conjugates **9-M5** and **9-M6** were detected in plasma, urine, and fecal samples. Their MS/MS spectra were dominated by the neutral loss of 80 Da. Metabolite **9-M3** was highly abundant in fecal samples. Its high-resolution mass spectra showed an [M+H]^+^ ion at *m*/*z* 257.1296, corresponding to the molecular formula C_15_H_16_N_2_O_2_. In the tandem mass spectra, **9-M3** produced fragment ions at *m*/*z* 243 ([M+H-CH_3_]^+^), *m*/*z* 215 ([M+H-CH_3_⋅-CO]^+^), and *m*/*z* 107 ([M+H-CH_3_-CO-C_7_H_9_N]^+^), indicating that it was methylated metabolite. The metabolic pathways of **9** are illustrated in Figure 4C.

#### Triterpenoids, Steroids, and Sesquiterpenoids

In addition to the major structural types (A) to (D), less abundant groups of compounds were also found in WDS in previous chemical studies (Kuang et al., 2009; Yang et al., 2014; Bhardwaj et al., 2016). They were taken into consideration in this study, and the representative scaffolds included triterpenoids (**32**, daturaolone), steroids (**41**, stigmasterol), and sesquiterpenoids (**20**, cinerolide). Identification of **32**, **41**, and **20** is depicted in Table 1.

When **32** was provided to rats, high amounts of prototype were detected in fecal samples. The methoxyl groups at C-23, -14, -29, or -30 may undergo hydroxylation, followed by glucuronide conjugation (Table 1; Sanchez-Gonzalez et al., 2015). The phase I and phase II metabolites of **41** were also observed *in vivo* samples. Among phase I metabolites, the hydroxylation products were extensively observed. This type of reaction involved the addition of one or two hydroxyl groups to the parent drug. The phase II biotransformation was mainly sulfation, and the phase II metabolite **41-M3** was formed by a reduction reaction followed by a sulfated reaction. Most steroids were metabolized in the same way as ganoderic acid D (Cheng et al., 2012). After 20 mg/kg oral administration, a large portion of **20** was metabolized. Although this compound was not observed in plasma samples, it occurred in the unchanged form in fecal samples. The UPLC/ESI/qTOF-MS analysis detected large proportions of three metabolites in plasma samples (Table 1). The high-resolution mass spectra of **20-M3** revealed a [M+H]^+^ signal at *m*/*z* 217.1211, indicating the molecular formula C_14_H_16_O_2_, corresponding to a demethylated derivative of **20**. Similar to cyperon (Duan et al., 2012), **20** presented fragment ions at *m/z* 231 ([M+H-O]^+^), 203 ([M+H-CO]^+^), and 190 ([M+H-C_3_H_5_]^+^) (Figure 4D). Two phase II metabolites of **20** were detected, namely, glucuronide conjugate (**20-M1**) and sulfate conjugate (**20-M2**) (Figure 4D), which disappeared when the sample was treated with β-glucuronidase.

### Characterization of WDS Metabolites at a Clinical Dosage

**FIGURE 5:**
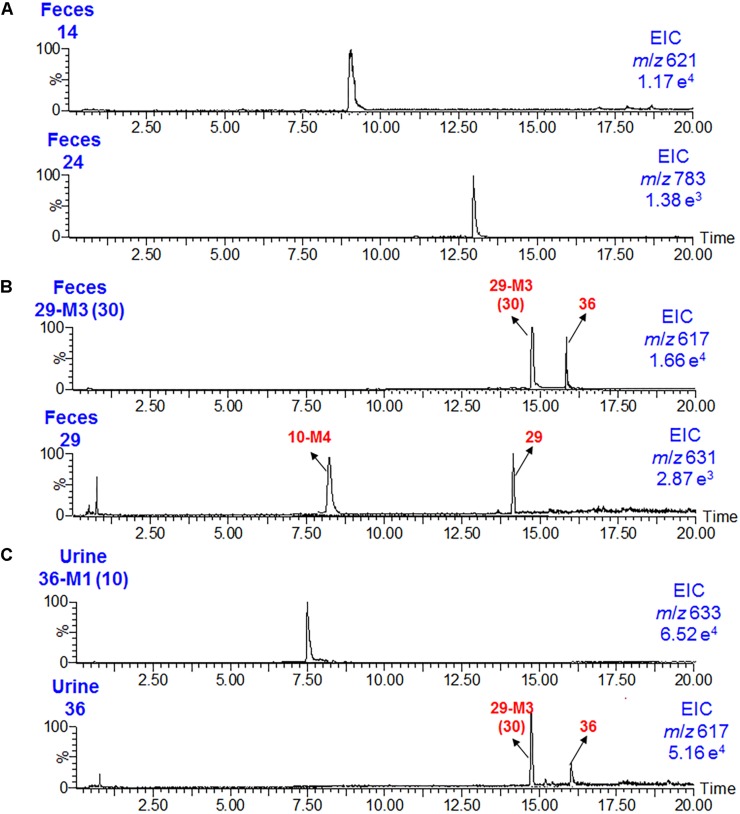
Ion chromatograms of **14** and **24** **(A)**, **29-M3** (**30**), and **29** **(B)** in rat fecal samples, and of **36-M1** (**10**) and **36** **(C)** in rat urine samples after the oral administration of the WDS.

To mimic traditional Chinese medicine clinical use, *D. metel* seeds were cooked in water to obtain the WDS extract. The dosage of WDS provided to rats (0.15 g/kg) was equivalent to the 1.5 g/day for a 60-kg human (Pharmacopoeia Commission, 2015). Under these conditions, 61 metabolites (including intact original compounds) were detected in rat plasma samples, 83 in urine samples, and 76 in fecal samples using qTOF-MS and LC/MS/MS. Withanolides, withanolide glucosides, and alkaloids were the major absorbed chemical components of WDS, and their oral bioavailability was as follows: withanolides >withanolide glucosides >amides and indoles. The contents of compounds **9**, **13**, and **20** are very high in WDS, and they could also be easily detected in WDS-administrated plasma samples. Interestingly, as shown in Table 1 and Figure 5, withanolide monoglucoside (**14**) appeared to have a better oral bioavailability than withanolide diglucoside (**24**). In addition, hydroxylation or (de)methylation was the most common reaction for withanolide glucosides. The absorption of the demethylated metabolite occurred at 7-OH (for instance **29-M3**, **30**) was easier than the prototypes (**29**); besides that, the hydroxylated metabolite at C-21 (**36-M1**, **10**) could be readily absorbed into circulation than compound *per se* (**36**) (Figures 4, 5).

Overall, and according to the metabolic pathways of single representative compounds, the major reactions of the WDS extract included phase II metabolism, hydroxylation, (de)methylation, and dehydrogenation. The phase II metabolites were confirmed by β-glucuronidase hydrolysis. The hydroxylation of **3**, **5**, **10**, **13**, **29**, **40**, and **41** was confirmed by rat liver microsomes incubation experiments, indicating that this hydroxylation was catalyzed by P450 enzymes. Withanolides and withanolide glucosides without a phenolic hydroxyl group at C-21 might readily undergo monohydroxylation. Compounds **9**, **10**, and **36** could add a methyl group, and compounds **20**, **21**, **28**, and **31** could lose a methyl group. Finally, five dehydrogenated metabolites (**1-M5**, **4-M2**, **10-M4**, **12-M5**, and **19-M3**) were characterized by LC/MS analysis (Table 1). Among the metabolic reactions described above, the phase II metabolism (glucuronidation or sulfation) was observed most commonly in the metabolism of WDS constituents.

## Conclusion

We propose the strategy “chemical analysis, metabolism of single representative compounds, and metabolism of the extract at the clinical dosage” to systematically characterize the *in vivo* metabolites of *D. metel* seeds. After the oral administration of *D. metel* seeds water extract to rats at a normal clinical dosage (0.15 g/kg), 113 phytochemicals (including intact original compounds) were identified or tentatively characterized by a highly sensitive LC/MS-MS method. As far as we know, this is the first report on the full metabolic profiling of the *D. metel* seeds water extract *in vivo*. This strategy could be helpful for pharmacokinetic studies of multi-component herbal products.

## Ethics Statement

The animal facilities and protocols were approved by the Animal Care and Use Committee of Harbin Medical University. All procedures were in accordance with the National Institutes of Health Guide for the Care and Use of Laboratory Animals (ILAR, 1996).

## Author Contributions

QW, BY, and CX participated in research design. CX, YL, HQ, LN, YZ, WL, XX, and YS conducted the experiments. QW, CX, and YL performed the data analysis.

## Conflict of Interest Statement

The authors declare that the research was conducted in the absence of any commercial or financial relationships that could be construed as a potential conflict of interest.
