# Supplementary material for: Characterization of the Metabolic Fate of Datura metel Seed Extract and Its Main Constituents in Rats
Source: Front Pharmacol. 2019 May 28;10:571. doi: 10.3389/fphar.2019.00571 (PMC6546908; doi:10.3389/fphar.2019.00571)
Supplement: Supplementary file 1 [file Data_Sheet_1.doc]

**Characterization of the metabolic fate of *Datura metel* seed extract and its main constituents in rats**

Cong Xia ^a,1^, Yan Liu ^b,1^, Hai Qi ^c^, Lulu Niu ^a^, Yuxuan Zhu ^a^, Wanying Lu ^a^, Xinyi Xu ^a^, Yongjian Su ^a^, Bingyou Yang ^b,*^, Qi Wang ^a,*^

**Affiliation:**

*^a^ Department of Medicinal Chemistry and Natural Medicine Chemistry, College of Pharmacy, Harbin Medical University,* *157 Baojian Road, Nangang District, Harbin 150081, China*

*^b^* *Key Laboratory of Chinese Materia Medica, Heilongjiang University of Chinese Medicine, Harbin 150040, China*

*^C^ Department of Cardiology, The 2^nd^ Affiliated Hospital of Harbin Medical University, Harbin 150001, China*

^*^. Corresponding author. Tel./fax: +86 045186660227.

*E-mail address:* mydearmumu@163.com (Q. Wang).

**Table of Contents**

**Figure S1.** Extracted ion chromatograms of **3-M3**, **5-M1**, and **13-M2** in rat liver microsomes (A), and in rats plasma after oral administration (B).

**Figure S2.** Characterization of the glucuronide conjugates in rats feces after oral administration of **1** and **40**. **1**, EIC of the mono-glucuronide conjugate in feces before (A) and after (B) *β*-glucuronidase hydrolysis and the reference standards (C); **40**, mono-glucuronide conjugate in feces before (D) and after (E) *β*-glucuronidase hydrolysis and the reference standards (F).

**Figure S3.** ^1^H NMR (400 MHz, DMSO-*d*_6_) spectrum of **1**.

**Figure S4.** ^13^C NMR (100 MHz, DMSO-*d*_6_) spectrum of **1**.

**Figure S5.** ^1^H NMR (400 MHz, DMSO-*d*_6_) spectrum of **3**.

**Figure S6.** ^13^C NMR (100 MHz, DMSO-*d*_6_) spectrum of **3**.

**Figure S7.** ^1^H NMR (400 MHz, DMSO-*d*_6_) spectrum of **5**.

**Figure S8.** ^13^C NMR (100 MHz, DMSO-*d*_6_) spectrum of **5**.

**Figure S9.** ^1^H NMR (400 MHz, DMSO-*d*_6_) spectrum of **9**.

**Figure S10.** ^13^C NMR (100 MHz, DMSO-*d*_6_) spectrum of **9**.

**Figure S11.** ^1^H NMR (400 MHz, DMSO-*d*_6_) spectrum of **18**.

**Figure S12.** ^13^C NMR (100 MHz, DMSO-*d*_6_) spectrum of **18**.

**Figure S13.** ^1^H NMR (400 MHz, DMSO-*d*_6_) spectrum of **22**.

**Figure S14.** ^13^C NMR (100 MHz, DMSO-*d*_6_) spectrum of **22**.

**Figure S15.** ^1^H NMR (400 MHz, DMSO-*d*_6_) spectrum of **29**.

**Figure S16.** ^13^C NMR (100 MHz, DMSO-*d*_6_) spectrum of **29**.

**Figure S17.** ^1^H NMR (400 MHz, DMSO-*d*_6_) spectrum of **32**.

**Figure S18.** ^13^C NMR (100 MHz, DMSO-*d*_6_) spectrum of **32**.

**Figure S19.** ^1^H NMR (400 MHz, DMSO-*d*_6_) spectrum of **40**.

**Figure S20.** ^13^C NMR (100 MHz, DMSO-*d*_6_) spectrum of **40**.

**Figure S21.** ^1^H NMR (400 MHz, DMSO-*d*_6_) spectrum of **41**.

**Figure S22.** ^13^C NMR (100 MHz, DMSO-*d*_6_) spectrum of **41**.

**Figure S1.** Extracted ion chromatograms of **3-M3**, **5-M1**, and **13-M2** in rat liver microsomes (A), and in rats plasma after oral administration (B).

**Figure S2.** Characterization of the glucuronide conjugates in rats feces after oral administration of **1** and **40**. **1**, EIC of the mono-glucuronide conjugate in feces before (A) and after (B) *β*-glucuronidase hydrolysis and the reference standards (C); **40**, mono-glucuronide conjugate in feces before (D) and after (E) *β*-glucuronidase hydrolysis and the reference standards (F).

**Figure S3.** ^1^H NMR (400 MHz, DMSO-*d*_6_) spectrum of **1**.

**Figure S4.** ^13^C NMR (100 MHz, DMSO-*d*_6_) spectrum of **1**.

**Figure S5.** ^1^H NMR (400 MHz, DMSO-*d*_6_) spectrum of **3**.

**Figure S6.** ^13^C NMR (100 MHz, DMSO-*d*_6_) spectrum of **3**.

**Figure S7.** ^1^H NMR (400 MHz, DMSO-*d*_6_) spectrum of **5**.

**Figure S8.** ^13^C NMR (100 MHz, DMSO-*d*_6_) spectrum of **5**.

**Figure S9.** ^1^H NMR (400 MHz, DMSO-*d*_6_) spectrum of **9**.

**Figure S10.** ^13^C NMR (100 MHz, DMSO-*d*_6_) spectrum of **9**.

**Figure S11.** ^1^H NMR (400 MHz, DMSO-*d*_6_) spectrum of **18**.

**Figure S12.** ^13^C NMR (100 MHz, DMSO-*d*_6_) spectrum of **18**.

**Figure S13.** ^1^H NMR (400 MHz, DMSO-*d*_6_) spectrum of **22**.

**Figure S14.** ^13^C NMR (100 MHz, DMSO-*d*_6_) spectrum of **22**.

**Figure S15.** ^1^H NMR (400 MHz, DMSO-*d*_6_) spectrum of **29**.

**Figure S16.** ^13^C NMR (100 MHz, DMSO-*d*_6_) spectrum of **29**.

**Figure S17.** ^1^H NMR (400 MHz, DMSO-*d*_6_) spectrum of **32**.

**Figure S18.** ^13^C NMR (100 MHz, DMSO-*d*_6_) spectrum of **32**.

**Figure S19.** ^1^H NMR (400 MHz, DMSO-*d*_6_) spectrum of **40**.

**Figure S20.** ^13^C NMR (100 MHz, DMSO-*d*_6_) spectrum of **40**.

**Figure S21.** ^1^H NMR (400 MHz, DMSO-*d*_6_) spectrum of **41**.

**Figure S22.** ^13^C NMR (100 MHz, DMSO-*d*_6_) spectrum of **41**.
